# Artificial‐intelligence‐driven discovery of prognostic biomarker for sarcopenia

**DOI:** 10.1002/jcsm.12840

**Published:** 2021-10-26

**Authors:** Heewon Chung, Yunju Jo, Dongryeol Ryu, Changwon Jeong, Seong‐Kyu Choe, Jinseok Lee

**Affiliations:** ^1^ Department of Biomedical Engineering, College of Electronics and Information Kyung Hee University Yongin‐si Gyeonggi‐do Republic of Korea; ^2^ Department of Molecular Cell Biology Sungkyunkwan University School of Medicine Suwon Republic of Korea; ^3^ Sarcopenia Total Solution Center Wonkwang University School of Medicine Iksan Republic of Korea; ^4^ Medical Convergence Research Center Wonkwang University Iksan Republic of Korea; ^5^ Department of Microbiology, and Institute of Wonkwang Medical Science Wonkwang University School of Medicine Iksan Jeonbuk Republic of Korea

**Keywords:** Sarcopenia, Muscle wasting, Artificial intelligence, Transcriptome, Diagnosis

## Abstract

**Background:**

Sarcopenia is defined as muscle wasting, characterized by a progressive loss of muscle mass and function due to ageing. Diagnosis of sarcopenia typically involves both muscle imaging and the physical performance of people exhibiting signs of muscle weakness. Despite its worldwide prevalence, a molecular method for accurately diagnosing sarcopenia has not been established.

**Methods:**

We develop an artificial intelligence (AI) diagnosis model of sarcopenia using a published transcriptome dataset comprising patients from multiple ethnicities. For the AI model for sarcopenia diagnosis, we use a transcriptome database comprising 17 339 genes from 118 subjects. Among the 17 339 genes, we select 27 features as the model inputs. For feature selection, we use a random forest, extreme gradient boosting and adaptive boosting. Using the top 27 features, we propose a four‐layer deep neural network, named DSnet‐v1, for sarcopenia diagnosis.

**Results:**

Among isolated testing datasets, DSnet‐v1 provides high sensitivity (100%), specificity (94.12%), accuracy (95.83%), balanced accuracy (97.06%) and area under receiver operating characteristics (0.99). To extend the number of patient data, we develop a web application (http://sarcopeniaAI.ml/), where the model can be accessed unrestrictedly to diagnose sarcopenia if the transcriptome is available. A focused analysis of the top 27 genes for their differential or co‐expression with other genes implied the potential existence of race‐specific factors for sarcopenia, suggesting the possibility of identifying causal factors of sarcopenia when a more extended dataset is provided.

**Conclusions:**

Our new AI model, DSnet‐v1, accurately diagnoses sarcopenia and is currently available publicly to assist healthcare providers in diagnosing and treating sarcopenia.

## Introduction

Ageing involves progressive changes in an individual's physiology. Sarcopenia, defined as muscle loss, is a leading cause of frailty in the elderly^1^, ^2^ and hence affects human health by potentiating disease occurrence leading to type 2 diabetes,^3^ heart and respiratory diseases and insufficiency in mechanical support.^3^, ^4^, ^5^, ^6^

The pathogenesis of sarcopenia is associated with ageing, which may impede either anabolic signalling to build components of the muscle, regenerative activity to repair damaged tissues, intricate circuits to deliver nervous signals in and out of the muscle, or cellular surveillance to maintain energy flexibility of the body.^7^, ^8^, ^9^, ^10^ In addition, malnutrition and immobility affect the onset and degree of sarcopenia.^11^, ^12^, ^13^ Such phenomena may occur individually or simultaneously to induce muscle atrophy when imbalanced or out of control, thereby resulting in causal complexity and difficulty in treating sarcopenia.^2^ Currently, no efficient drug for treating sarcopenia is available,^2^, ^7^ although efforts have been expended to identify biomarkers for early diagnosis to develop preventive medicines or alleviate sarcopenia. A recent study using multi‐ethnic aged muscle biopsies exemplifies the nuclear‐encoded mitochondrial genes as a typical gene set that can be applied across ethnicities.^14^ In addition, serum biomarkers that can reliably predict sarcopenia has been reported.^15^ Candidate biomarkers were selected from the literature, followed by comparative biochemical analyses of sera between sarcopenia and normal elderly groups. Four combined biomarkers indicated higher diagnostic accuracy for sarcopenia than individual biomarkers, thereby re‐emphasizing the causal complexity contributing to sarcopenia. Therefore, the identification of additional biomarkers that reliably reflect sarcopenia may warrant early diagnosis to facilitate the deployment of preventative medicine and early intervention therapy.^16^

In this study, we aimed to develop an artificial intelligence (AI) model for sarcopenia diagnosis using a previously published transcriptome dataset that contain differentially expressed genes in muscle biopsies from patients with sarcopenia and age‐matched healthy individuals across three ethnic groups. The AI model developed in this study yielded a diagnostic accuracy of >94%, which is an unprecedented level. To the best of our knowledge, our current study is the first attempt to develop an AI model to diagnose sarcopenia based on a transcriptome dataset only.

## Materials and methods

### Datasets

We used a published transcriptomic dataset deposited in the Gene Expression Omnibus of the National Center for Biotechnology (https://www.ncbi.nlm.nih.gov/geo/) under accession number GSE111017.^14^ (*Table* S1) summarizes the subject information from the dataset. A total of 118 subjects (mean age, 73.34 ± 5.43) participated in this study, of whom 86 were healthy and 32 were sarcopenic. Subjects of three different races from different studies participated in the study: 40 subjects from the Hertfordshire sarcopenia study (HSS; 36 healthy vs. 4 sarcopenic), 39 from the Jamaica sarcopenia study (JSS; 30 healthy vs. 9 sarcopenic) and 39 from the Singapore sarcopenia study (SSS; 20 healthy vs. 19 sarcopenic). Transcriptome analysis includes 17 339 genes, which can be potential biomarkers for the diagnosis of sarcopenia (*Data* S1).

For hold‐out validation, we segmented the data into training (80%) and testing (20%) datasets in a stratified manner based on both the race (HSS, JSS, and SSS) and outcome (normal and sarcopenic). Accordingly, we used 94 subjects as the training dataset (69 normal and 25 sarcopenic) and 24 data as the testing dataset (17 normal and 7 sarcopenic). The testing dataset was isolated and used only to evaluate the performance of the proposed AI model. For box or scatter plots, data are shown as median ± interquartile. *P < 0.05, **P < 0.01, ***P < 0.001; *P* values calculated using either two‐tailed Wilcoxon rank sum test or two‐way ANOVA followed by Tukey's multiple comparisons test as described. This study was approved by Institutional Review Board Wonkwang University (WKIRB‐202108‐SB‐060).

### Preprocessing

In the dataset, each of the 17 339 genes can be the feature for the AI model. Some features (gene information) were missing from the training and testing datasets (*Supporting information*, *Figure* S1). To manage the missing features, we calculated the mean value from the training dataset for each feature and replaced the missing feature with the mean value in both the training and testing datasets. Subsequently, we standardized the dataset, which is a typical requirement for machine learning algorithms. The standardization changes the data distribution of each feature with a mean of zero and a standard deviation of one.

(1) $${\text{Data}}_{\text{standard}}=\frac{\text{Data}-\text{mean}\left(\text{train}\right)}{\mathrm{SD}\left(\text{train}\right)}\;,$$

where *mean*(*train*) and *SD*(*train*) are the mean and standard deviation values, respectively, for each feature from the training dataset. Standardization was applied to both the training and testing datasets.

### Feature selection

To select important features that affect clinical severity, we investigated the contribution of each of the 17 339 input variables on sarcopenia diagnosis via feature importance analysis using a random forest (RF),^17^ extreme gradient boosting (XGBoost),^18^ and adaptive boosting (AdaBoost)^19^, ^20^ algorithms.

By repeating the five‐fold cross‐validation 10 times, we obtained the best hyperparameters. For AdaBoost, we set the hyperparameters as follows: number of tree estimators, 200; learning rate, 0.2. For the RF, we set the number of tree estimators to 100, maximum depth to four and maximum features to five. For XGBoost, we set the maximum depth to four, learning rate to 0.1, number of tree estimators to 100, regularization parameter α to 1.0, fraction of observations to 0.9 and fraction of columns to 0.9.

Based on the resultant 50 sets of feature importance values for each classifier (AdaBoost, RF and XGBoost), we averaged the values and normalized them such that the importance values from each classifier ranged from zero to one. Next, we averaged the importance values for the final ranked feature importance values. Finally, we determined the optimal number of top features to be incorporated into the AI diagnosis model based on the cross‐validation results.

### Development of artificial intelligence model based on deep neural network

A deep neural network (DNN) was used to develop the final AI model for sarcopenia diagnosis. In the DNN approach, we investigated up to five hidden layers, and each layer depth (node) up to the previous layer depth (node). For the input layer, we first ranked the features based on their importance and increased the number of top features used in the input layer. Accordingly, we used the top 27 features as input layers. For the fully connected (FC) layers as hidden layers, we applied dropouts by changing the dropout rate from 0 to 0.5, at increments of 0.1. The last FC layer was fed into a sigmoid layer, which is an output layer that provides the probabilities of patient severity. We trained the models using the ADAM optimizer and binary cross‐entropy cost function with a learning rate of 0.0001 and a batch size of 64.

For each set of top features, we obtained the best cross‐validation accuracy using two metrics, that is, the area under the curve and the balanced accuracy (Equation 2).

(2) $$\text{Balanced}\;\text{Accuracy}=\frac{\text{Sensitivity}+\text{Specificity}}{2\;}$$

**Figure 1:**
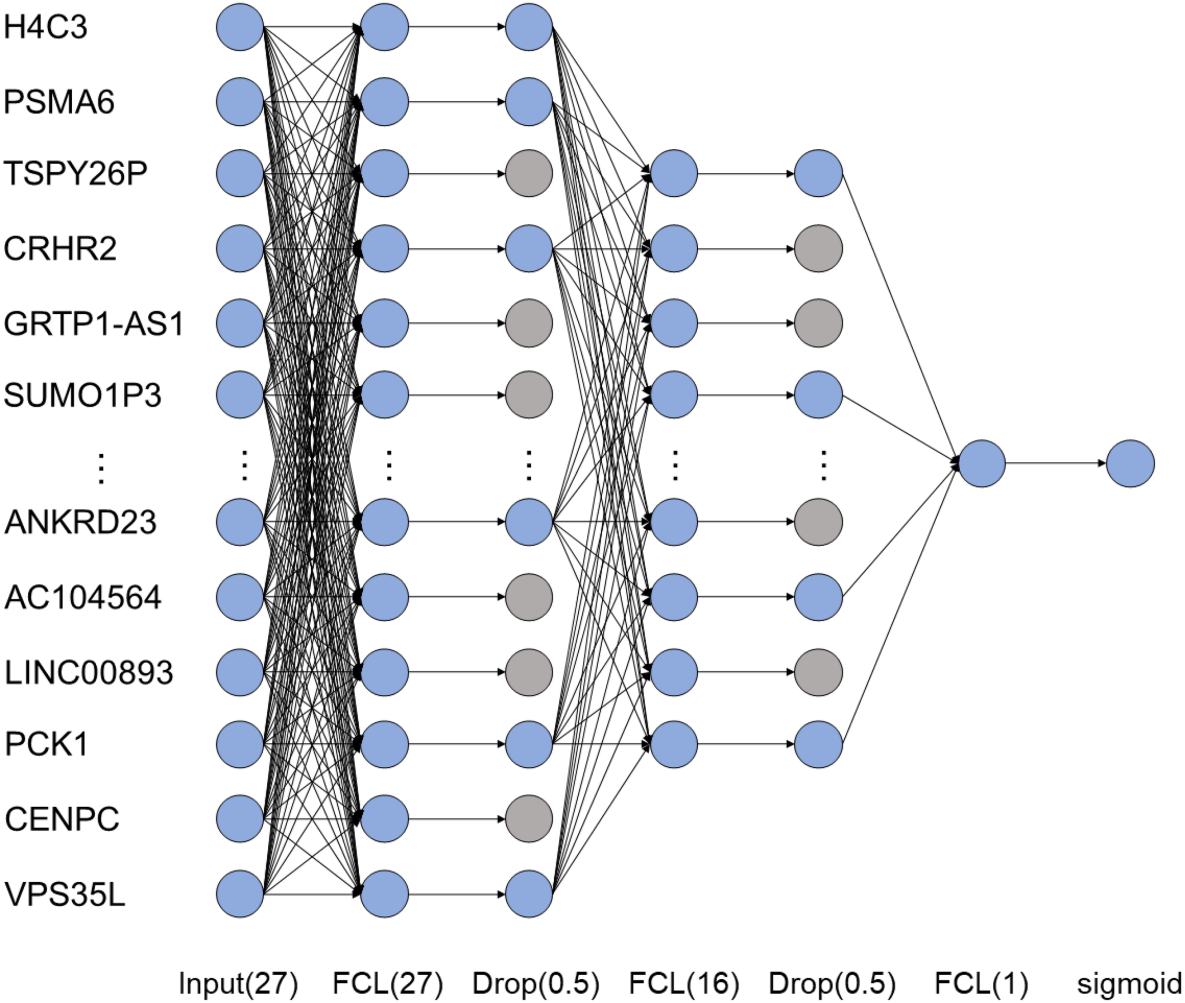
Proposed DSnet‐v1 with four‐layer deep neural network (DNN) for the diagnosis of sarcopenia.

Based on the cross‐validation accuracy analysis, we modelled a four‐layer DNN using the top 27 features, as shown in (*Figure* 1). The four‐layer DNN comprised an input layer, two FC layers as hidden layers, and an output layer. The input layer was fed into a series of two FC layers comprising 27 and 8 nodes, respectively. In the two FC layers, we used a dropout rate of 0.5. Subsequently, the last FC layer was fed into the sigmoid layer. Our proposed DNN model was named DSnet‐v1, which represents the DNN for sarcopenia diagnosis version 1.

### Implementation

We implemented and trained the DNN using TensorFlow (version: tensorflow‐gpu 2.0), whereas we used NumPy (version: 1.16.4), Pandas (version: 0.25.3), Matplotlib (version 3.1.2) and Scikit‐learn (version 0.22.1) to build the model and analyse the results. We trained the models using the ADAM optimizer and the binary cross‐entropy cost function shown in Equation 3 by adjusting the learning rate to 0.0005 and 0.0001 and using a batch size of 64 on an NVIDIA GeForce GTX 1080 Ti GPU.

(3) $$\mathrm{BCE}\left(x\right)=-\frac{1}{N}\sum_{i=1}^{N}y_{i}\log\left(p\left(y_{i}\right)\right)+\left(1-y_{i}\right)\log\left(1-p\left(y_{i}\right)\right),\;$$

where *y*_*i*_ is the label (1 for sarcopenia and 0 for normal) and *p*(*y*_*i*_) is the classified probability of each subject being sarcopenic for a batch size comprising *N* patients.

### Performance evaluation of AI models

To evaluate the performance of DSnet‐v1 for the diagnosis of sarcopenia, we used accuracy metrics of sensitivity, specificity, accuracy, balanced accuracy and area under the receiver operating characteristics (AUROC).

In the training dataset, our DSnet‐v1 was evaluated based on 5‐fold stratified cross‐validation. Subsequently, the diagnostic performance was evaluated independently using an isolated testing dataset. To compare the performance of DSnet‐v1 with those of other external AI models, we separately trained the models of RF, XGBoost and AdaBoost, each of which was evaluated with hyperparameter search.

### Box plots, correlation and network analysis

The evaluation and visualization of gene expression (boxplots), Spearman's correlation (correlogram matrices), and gene network for 27 AI‐featured genes and their associated genes were conducted using R packages ggpubr, ggplot2, igraph, ggraph, egg, corrr, corrplot, dplyr, tidyverse, and reshape (https://www.r‐project.org). The network visualization of human phenotype ontology was assessed using Enrichr.^21^

### Public website deployment

We deployed DSNet‐v1 on a public web server (http://sarcopeniaAI.site/) through Amazon Web Services (AWS), which provides secure, durable and scalable service. After accessing the website, a user enters the 27 genes, which are encoded to the website server and can immediately obtain the diagnosis result of sarcopenia. There is no need to enter any private information other than gene information, and the entered information is immediately deleted when the diagnosis result is derived, so there is no risk of information exposure.

## Results

### Feature selection and cross‐validation

(*Table* 1) summarizes the results of the ranked feature importance from the RF, XGBoost, and AdaBoost, as well as their combination: We ranked the top 27 features based on the combination among the 17,339 features. The RF results indicated that *GRTP1‐AS1* possessed the highest importance value, followed by *SUMO1P3*, *TEX261*, *SMIM26*, and *H4C3*. The XGBoost results indicated that *H4C3* possessed the highest importance value, followed by *PSMA6*, *AC002070.1*, *PCK1*, and *CENPC*. The AdaBoost results indicated that *TSPY26P* possessed the highest importance value, followed by *STAG3L3*, *CRHR2*, *PEF1*, and *FKBP1C*. By averaging the values obtained from the three models, *H4C3* exhibited the highest importance value, followed by *PSMA6*, *TSPY26P*, *CRHR2*, and *GRTP1‐AS1*. The full list of ranked feature importance values is summarized in (Data S2).

**Table 1:** Feature importance

Rank	Feature name	Gene name/ensembl gene ID	Random forest	XGBoost	AdaBoost	Mean
1	H4C3	H4 clustered histone 3	0.5715	1.0000	0.2857	0.6191
2	PSMA6	Proteasome subunit alpha 6	0.2263	0.8000	0.4286	0.4850
3	TSPY26P	Testis specific protein, Y‐linked 26, pseudogene	0.0000	0.0667	1.0000	0.3556
4	CRHR2	Corticotropin releasing hormone receptor 2	0.0943	0.2000	0.7143	0.3362
5	GRTP1‐AS1	Growth hormone regulated TBC protein 1‐antisense	1.0000	0.0000	0.0000	0.3333
6	SUMO1P3	SUMO1 Pseudogene 3	0.9620	0.0000	0.0000	0.3207
7	STAG3L3	Stromal antigen 3‐like 3, transcribed_unprocessed_pseudogene	0.0000	0.0000	0.8571	0.2857
8	KAT2A	Lysine acetyltransferase 2A	0.4174	0.0000	0.4286	0.2820
9	PEF1	Penta‐EF‐hand domain containing 1	0.0000	0.0667	0.7143	0.2603
10	SMIM26	Small integral membrane protein 26	0.5975	0.1333	0.0000	0.2436
11	FKBP1C	FKBP prolyl isomerase family member 1C	0.0000	0.0000	0.7143	0.2381
12	TEX261	Testis expressed 261	0.7084	0.0000	0.0000	0.2361
13	PFKFB4	6‐Phosphofructo‐2‐kinase/fructose‐2,6‐biphosphatase 4	0.2055	0.3333	0.1429	0.2272
14	AC116913.1	No NCBI gene ID yet, novel noncoding transcript, antisense to MAP 2 K1 and SNAPC5/ENSG00000261351	0.2651	0.4000	0.0000	0.2217
15	TBC1D8	TBC1 domain family member 8	0.0690	0.0000	0.5714	0.2135
16	MYF5	Myogenic factor 5	0.0000	0.3333	0.2857	0.2063
17	TPSAB1	Tryptase alpha/beta 1	0.1825	0.0000	0.4286	0.2037
18	AC002070.1	LOC105370027/ENSG00000248636	0.0000	0.4667	0.1429	0.2032
19	RASSF1	RAS association domain family member 1	0.3554	0.2000	0.0000	0.1851
20	AC006971.1	No NCBI gene ID yet, novel noncoding transcript, ARHGAP5 pseudogene/ENSG00000218586	0.0000	0.2667	0.2857	0.1841
21	SNX12	Sorting nexin 12	0.4790	0.0000	0.0000	0.1597
22	ANKRD23	Ankyrin repeat domain 23	0.0000	0.3333	0.1429	0.1587
23	AC104564.5	No NCBI gene ID yet, novel noncoding transcript/ENSG00000265625	0.0430	0.0000	0.4286	0.1572
24	LINC00893	Long intergenic non‐protein coding RNA 893	0.0430	0.0000	0.4286	0.1572
25	PCK1	Phosphoenolpyruvate carboxykinase 1	0.0000	0.4667	0.0000	0.1556
26	CENPC	Centromere protein C processed_pseudogene	0.0000	0.4667	0.0000	0.1556
27	VPS35L	*VPS35* endosomal protein sorting factor like	0.1680	0.0000	0.2857	0.1512

**Figure 2:**
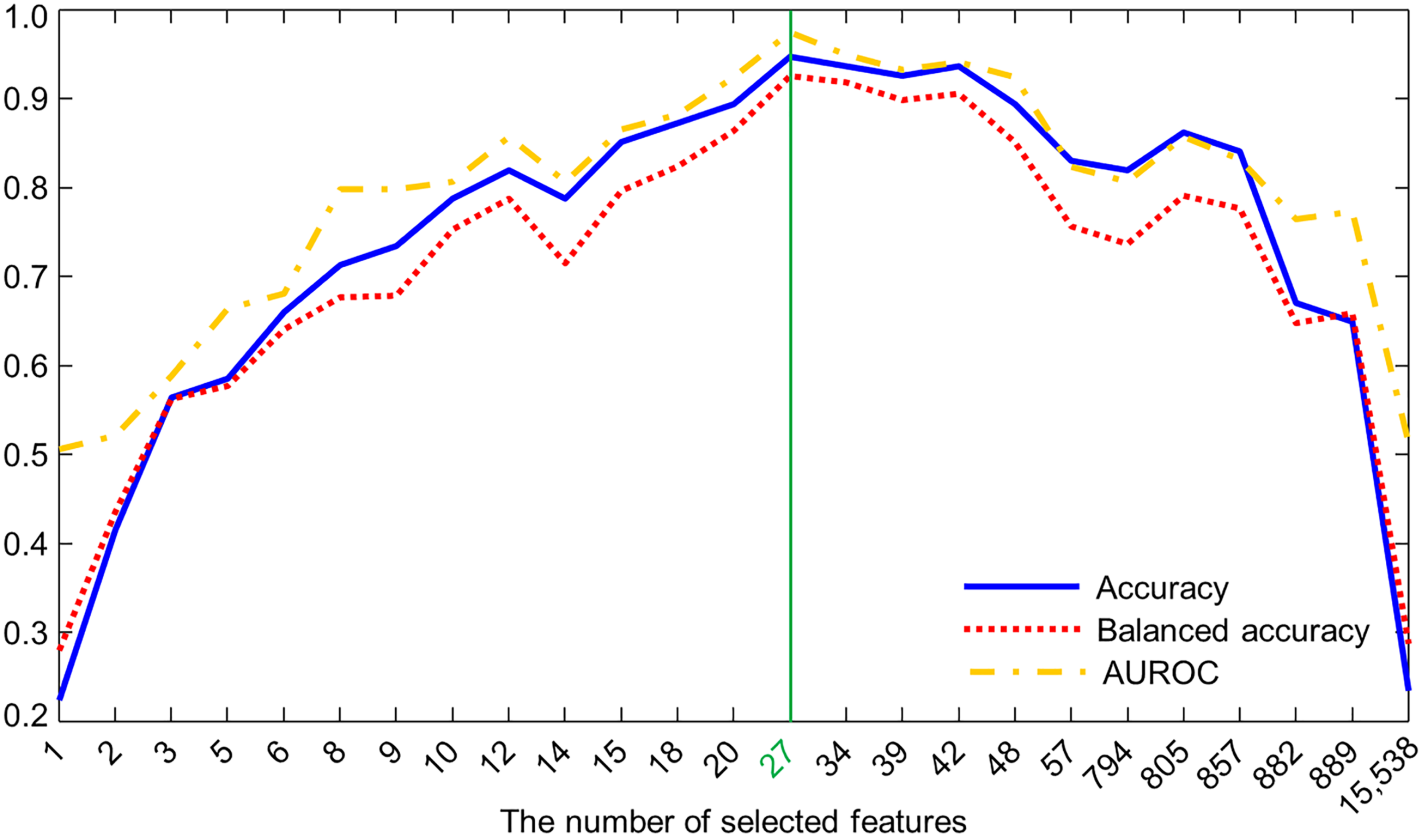
Accuracy, balanced accuracy and area under the receiver operating characteristics (AUROC) according each different number of selected top features.

We investigated the cross‐validation performance using metrics of sensitivity, specificity, accuracy, balanced accuracy, and AUROC. (*Figure* 2) shows the values of accuracy, balanced accuracy, and AUROC based on each number of selected top features. Detailed results are summarized in (*Table* S2). For the accuracy metrics, we identified the optimal DNN, including the hyperparameters for each selected feature. The results show that all the accuracy metrics, that is, accuracy, balanced accuracy and AUROC, increased as the number of selected features increased until 27. As the number of selected features exceeded 27, all accuracy metrics decreased as the number of selected features increased. For the top 27 features, we obtained a sensitivity of 0.88, specificity of 0.97, accuracy of 0.95, balanced accuracy of 0.93, and AUROC of 0.97.

(*Table* 2) summarizes the comparison of the cross‐validation accuracy. The results show that DSnet‐v1 provided the highest values of all accuracy metrics.

**Table 2:** Comparison of cross‐validation evaluation metrics (mean ± standard deviation)

Model	Cross‐validation results			
	Sensitivity	Specificity	Accuracy	Balanced accuracy (%)
RF	0.7448 ± 0.15832	0.8790 ± 0.1441	0.8503 ± 0.0.096	0.8119 ± 0.0813
XGBoost	0.7162 ± 0.1866	0.86485 ± 0.1415	0.8292 ± 0.1153	0.7905 ± 0.1014
AdaBoost	0.7848 ± 0.1705	0.8971 ± 0.0674	0.8719 ± 0.0487	0.8418 ± 0.0593
DNN	0.8772 ± 0.1072	0.9825 ± 0.0317	0.9583 ± 0.0439	0.9286 ± 0.0596

DNN, deep neural network; RF, random forest.

### Performance of proposed DNN

Using the isolated testing dataset (*n* = 4), DSnet‐v1 showed a sensitivity of 1.00, specificity of 0.94, accuracy of 0.96, balanced accuracy of 0.97, and AUROC of 0.99. (*Table* 3) shows the diagnostic performances of various AI models; as shown, our proposed four‐layer DNN provided a higher accuracy, more balanced accuracy, and higher AUROC values than the other external AI models: the RF, XGBoost, and AdaBoost. (*Figure* *S2*) shows the ROC curves for model comparison.

**Table 3:** Comparison of prediction performances among prediction models in test dataset

Model	TN	FP	FN	TP	Sensitivity	Specificity	Accuracy	Balanced accuracy	AUROC
RF	13	4	1	6	0.8571	0.7647	0.7917	0.8109	0.7479
XGBoost	12	5	1	6	0.8571	0.7059	0.7500	0.7815	0.7563
AdaBoost	14	3	1	6	0.8571	0.8235	0.8333	0.8403	0.8319
DNN	16	1	0	7	1.0000	0.9412	0.9583	0.9706	0.9916

AUROC, area under the receiver operating characteristics; DNN, deep neural network; RF, random forest.

**Figure 3:**
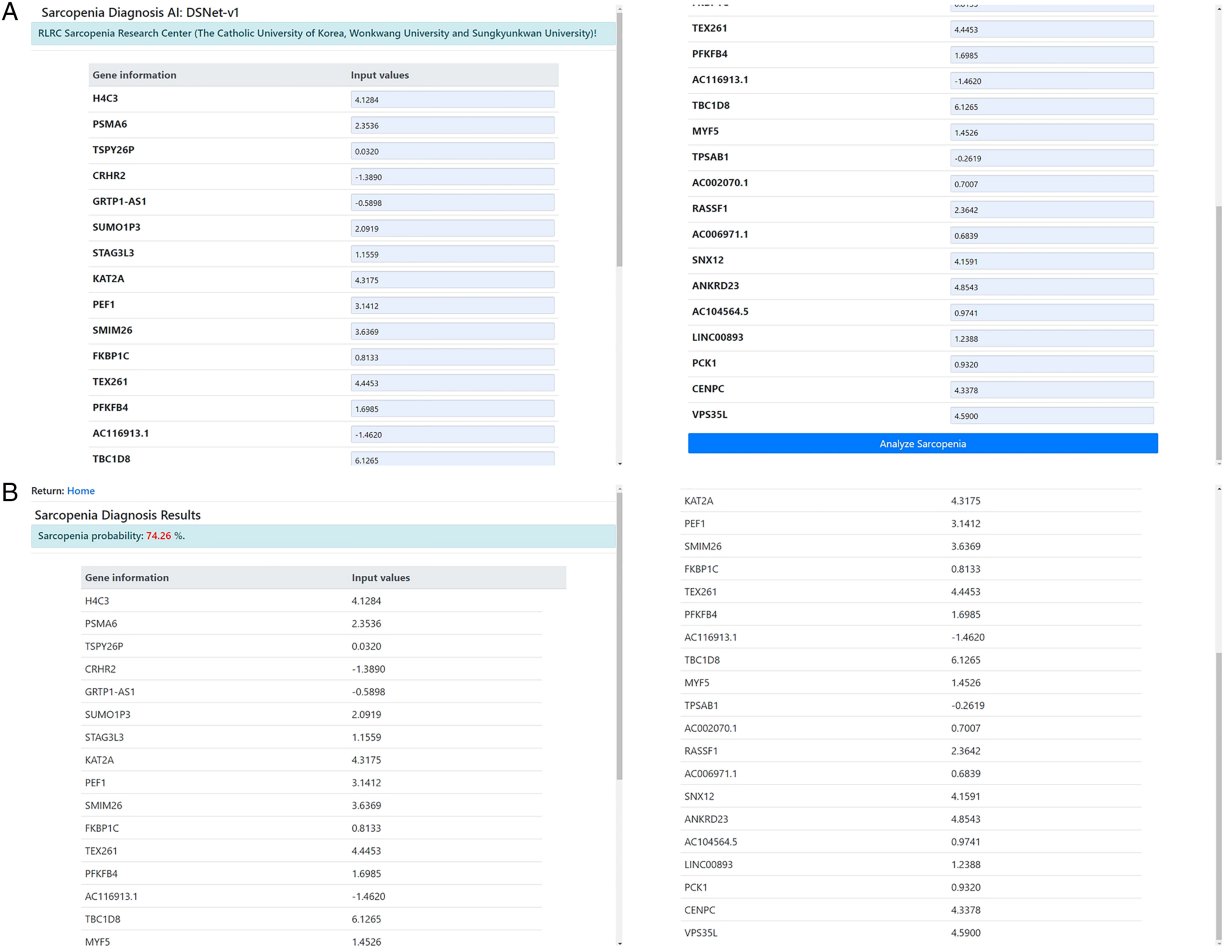
Developed AI model (DSNet‐v1) was successfully deployed on a public website (http://sarcopeniaAI.ml/)

The web application provides the probability of sarcopenia, as shown in (*Figure* 3). A user inputs his or her quantized gene information (as shown in *Figure* 3A), and the diagnosis results are presented (as shown in *Figure* 3B).

### Biological relevance of 27 AI‐featured genes

**Figure 4:**
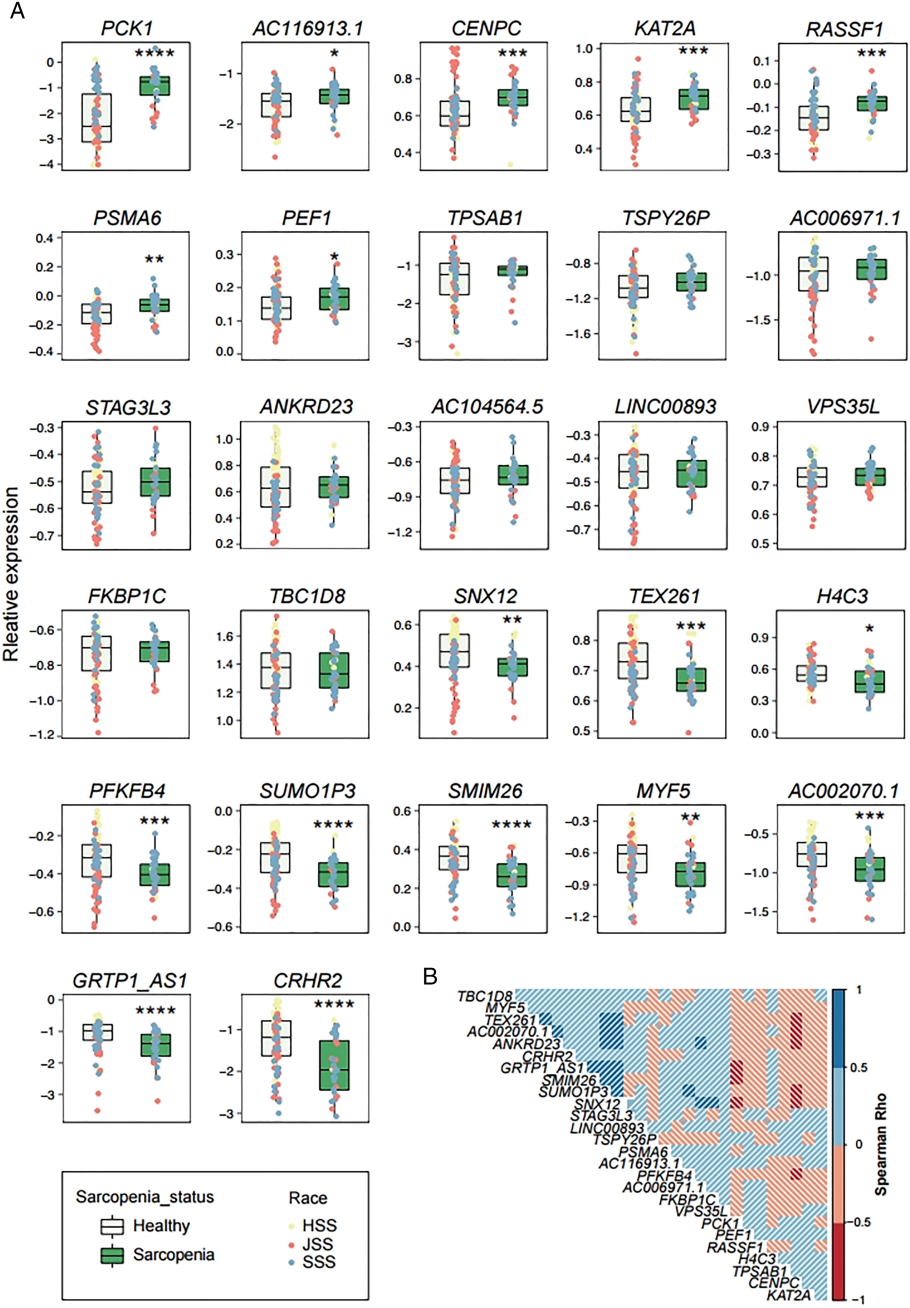
The visualization of expression and correlation of 27 AI‐featured gene. (A) Boxplots showing the relative expression of each gene in the healthy (white box) and sarcopenic elderly (green box). Yellow (HSS), pink (JSS) or blue (SSS) dots represent the gene expression of each individual subject in three races. Data are median ± interquartile. *P < 0.05, **P < 0.01, ***P < 0.001; *P* values calculated using two‐tailed Wilcoxon rank sum test. (B) Correlogram matrices display Spearman's rho between the genes facing each side of the square. The depth of the shading at the correlation matrices indicates the magnitude of the correlation as shown in the scale.

We evaluated the gene expression of 27 selected features (genes) in patients with sarcopenia (green box) and in age‐matched healthy individuals (white box) (*Figure* 4A). The gene expression of each individual for the three races (HSS, JSS and SSS) is indicated by red, blue and green dots, respectively. As shown in (*Figure* 4), the expression of seven genes (*PCK1*, *AC116913.1*, *CENPC*, *KAT2A*, *RASSF1*, *PSMA6* and *PEF1*) was elevated in the sarcopenic muscle, whereas 10 genes (*SNX12*, *TEX261*, *H4C3, PFKFB4*, *SUMO1P3*, *SMIM26*, *MYF5*, *AC002070.1*, *GRTP1‐AS1* and *CRHR2*) showed reduced expression. The remaining 10 genes did not show statistical differences between patients with sarcopenia and age‐matched healthy individuals. Twenty‐seven AI‐featured genes were positively (blue) or negatively (red) correlated with each other (the depth of colour for Spearman's rho between 0.0 and 0.5) (*Figure* 4B).

**Figure 5:**
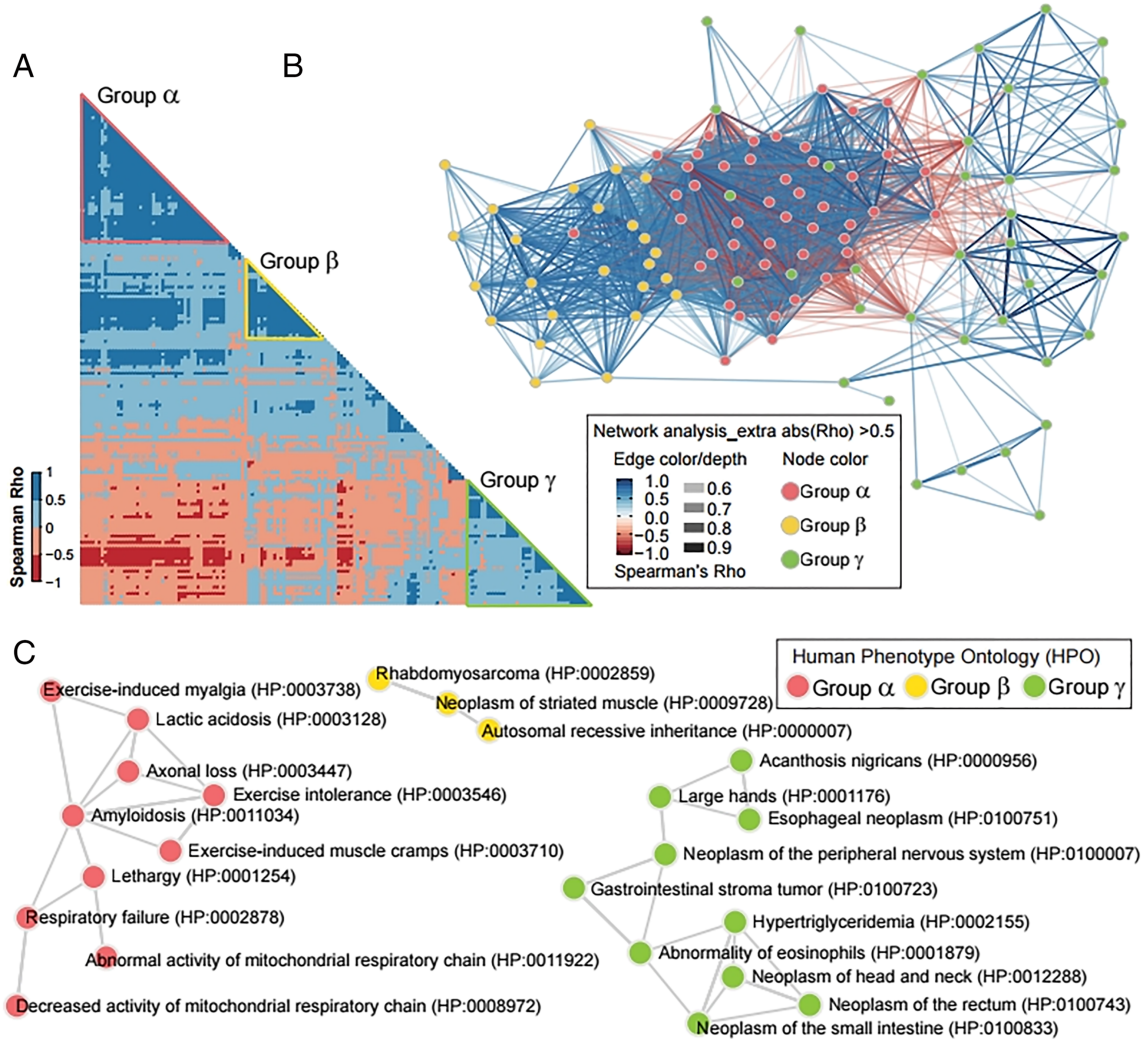
Co‐expression and human phenotype ontology assay categorizing 27 artificial intelligence (AI)‐featured genes into three groups related with skeletal muscle function, metabolism and diseases. (A) Correlogram matrices display Spearman's rho of two genes facing each side of the square. The shading intensity of the correlation matrices displays Spearman's Rho as presented in the scale (left‐hand side of the correlogram). The red, yellow and green triangles on the correlogram tie group α, β and γ, respectively. (B) Gene network showing co‐expression of group α, β and γ. The Spearman's rho of two node (gene) generates the colour and depth of each edge. The colour (pink, yellow and green) of node indicates each group (group α, β and γ). The gene symbols are indicated in *Figure* S2. (C) Three networks generated by Enrichr showing Human Phenotype Ontology that associated with each group. The colour of nodes (HPO terms) indicates each group and the edge means sharing common genes.

For a better understanding, we performed a transcriptome‐wide co‐expression analysis. We selected the top five correlative transcripts from each of the 27 featured genes and visualized them using a correlogram matrix (*Figure* 5A) and a gene network (*Figure* 5B). Three distinguishable groups (α, β and γ) were generated from Spearman's rank correlation coefficient (rho). Groups α and β generated geologically confined regions from other genes with a high positive correlation in each group (Spearman's rho between 0.5–1.0). Although group γ failed to form a highly associated cluster, unlike groups α and β, it was negatively associated (red edges) in both groups. Furthermore, three groups (*Figure* 5A,B) were generated via network analysis. Finally, the human phenotype ontology (HPO) (https://hpo.jax.org/) assay revealed the physiological and pathological relevance of the three groups (*Figure* 5C). The genes assigned to group α were linked to the HPO of exercise‐induced myalgia (HP:0003738), exercise intolerance (HP:0003546), exercise‐induced muscle cramps (HP:0003710), amyloidosis (HP:0011034), lactic acidosis (HP:0003128), axonal loss (HP:0003447), respiratory failure (HP:0002878) and mitochondrial respiratory chain (HP:0008972). Group β was associated with the HPO of rhabdomyosarcoma (HP:0002859), neoplasm of striated muscle (HP:0009728) and autosomal recessive inheritance (HP:0000007). Group γ comprised genes associated with the HPO of acanthosis nigricans (HP:0000956), oesophageal neoplasm (HP:0100751), gastrointestinal stromal tumour (HP:0100723), abnormality of eosinophils (HP:0001879), hypertriglyceridemia (HP:0002155), neoplasm of the small intestine (HP:0100833), large hands (HP:0001176), neoplasm of the small intestine (HP:0100833), neoplasm of the head and neck (HP:0012288) and neoplasm of the peripheral nervous system (HP:0100007). The seemingly separated group γ from groups α and β might be due to either a variable clinical history of sarcopenia patients or an as‐yet unknown biological interaction affecting sarcopenia pathogenesis. Further studies based on an extended dataset will be necessary to understand the co‐expression gene features comprehensively.

## Discussion

Our proposed AI model, DSNet‐v1, successfully diagnosed sarcopenia accurately (100% sensitivity, 94.12% specificity, 95.83% accuracy, 97.06% balanced accuracy, and 0.99 AUROC). This model presents several unique characteristics. First, our model was developed based on subjects across three different continents: Europe, Africa and Asia. Second, our AI diagnosis model was developed using a vast amount of gene information (i.e. 17 339 genes), and feature importance analysis was performed to identify genes associated with sarcopenia. Finally, we created a web application (http://sarcopeniaAI.ml/) to access the model. We believe that allowing the public to access the AI model will facilitate the validation and improvement of the model.

We observed that the expression patterns of several genes varied among the three races (*Figure* 4A). For instance, the *CENPC* expression was elevated in the sarcopenia of SSS and HSS, but not in that of JSS. The expression of *TEX261* reduced significantly in the sarcopenia of HSS and JSS, but not in that of SSS. This implies that regardless of race, certain factors might contribute to or be related to sarcopenia. Additionally, some other factors may be race‐specific sarcopenic factors. Indeed, we observed a race‐specific alteration of 27 genes in sarcopenic muscle compared to the healthy (*Figure* S3). For instance, *AC104564.5*, *TPSAB1*, *PFKFB4* and *GTTP1‐AS1* were altered only in the HSS. *AC116913.1* (a.k.a. *Lnc‐SNAPC5‐1*), *RASSF1*, *PSMA6* and *CRHR2* were changed only in the JSS, whereas *CENPC*, *KAT2A*, *PEF1*, *VPS35L*, *H4C3* and *SUMO1P3* were altered only in the SSS. Although it might imply that there is a race‐specific pathology in sarcopenia, the power (n) might be not enough to lead to a clear conclusion. In the case of *PCK1*, it failed to generate a statistical significance in the HSS. However, the mean expression was escalated. Because *PCK1* is known to be expressed in only gluconeogenic or glyceroneogenic tissues such as the liver, kidney, intestine and fat cells (https://www.gtexportal.org/), the altered expression of *PCK1* might imply the anatomical loss of muscle and gain of adipocytes. Besides, Migliavacca *et al*. clearly demonstrated that gene sets associated with mitochondrial function and NAD^+^ biosynthesis pathway were tightly associated with the development/progression of sarcopenia.^14^ Although our AI‐featured 27 genes did not include a gene directly involved mitochondrial and NAD^+^ biosynthesis metabolism, several genes could be associated indirectly with two pathways (i.e. mitochondria and NAD^+^ biosynthesis). For instance, it is well demonstrated that NAD^+^ biosynthesis and mitochondria function are tightly associated with sirtuin, a NAD^+^‐dependent protein deacetylase^22^, ^23^ and the protein acetyltransferase could be a count partner.^24^ KAT2A is one of the well‐defined count partners of Sirtuins^25^ and a very recent study proposed that KAT2A regulates muscle integrity.^26^ In addition, the gene‐regulating glycolysis displayed altered gene expression. An additional intensive study of these 27 genes featured by our AI algorithm will expand our understanding of muscle ageing and sarcopenia.

Using Spearman's correlation assay, we identified three functional groups that were highly associated with 27 AI‐featured genes (*Figure* 5). Interestingly, groups α and β were associated with HPO terms of skeletal muscle function (e.g., exercise, lactate metabolism and abnormal mitochondrial function) and diseases with muscular symptoms (e.g., amyloidosis, axonal loss and rhabdomyosarcoma). Sarcopenia is often associated with physical frailty,^23^ reduced muscle function,^27^ and mitochondrial dysfunction.^14^ In addition, recent studies revealed that amyloidosis in skeletal muscle is associated with mitochondrial dysfunction^28^, ^29^ and muscle diseases including inclusion body myositis,^30^, ^31^ indicating that both mitochondrial dysfunction and amyloidosis may trigger sarcopenia. In group γ, which generated a negative cluster against both groups α and β, most of the HPO terms were related to neoplasms. One HPO term was hypertriglyceridemia (HPO:0002155), which is known to be associated with sarcopenic obesity.^32^

Our study has several limitations. First, to find the 27 genes, we used the three machine learning algorithms such as RF, XGBoost and AdaBoost, which result in opposing feature importance values for some genes. To improve generalization performance, we used an ensemble approach, which combines machine learning techniques into one stable model by reducing variance and bias. To investigate the effectiveness, we compared the performance when the selected features are obtained from RF, XGBoost, AdaBoost and their ensemble in *Table* S3. The results show that the ensemble approach provided the highest accuracy metrics. It implies that the ensemble may help to improve generalization performance. However, for the combination, we equally weighted the feature importance values. In the future work, we will further investigate the ways to combine the values more efficiently and accurately. Second, our proposed AI diagnosis model was validated using an isolated test dataset (*n* = 24), which was a dataset segregated from an entire dataset. It may be necessary to validate our AI model using external datasets, such as prospectively collected data. In addition, we plan to further develop DSnet‐v1 and change its name to DSnet‐v2 and DSnet‐v3. To update the model, we will use our developed web application to acquire additional data and validate the model. Currently, the application does not store any information entered by users. However, we plan to store information entered by users upon agreement to improve the AI model via a real‐time learning process. Third, our data included only three subjects of different races. In future studies, we will train and apply our AI model to more datasets comprising more diverse subjects.

## Conclusion

Our AI model with 27 selected genes diagnosed sarcopenia accurately. We believe that it can facilitate healthcare providers in treating patients with early‐stage sarcopenia.

## Conflict of interest

Heewon Chung, Yunju Jo, Dongryeol Ryu, Changwon Jeong, Seong‐Kyu Choe and Jinseok Lee declare that they have no conflict of interest.

## Supporting information


**Figure S1.** Histogram for the number of available gene information. 39 subjects include approximately 16,100 gene information out of 17,339. 40 subjects include approximately 16,600 gene information. 39 subjects include approximately 16,700 gene information.
**Figure S2.** Receiver operating characteristic curves (ROCs); four different models of RF, XGBoost, AdaBoost, and DSnet‐v1based on testing data.
**Figure S3.** Scatter plots showing the relative expression of each gene in the healthy and sarcopenic elderly in the three different races.
**Figure S4.** Gene network showing co‐expression of group α, β, and γ. This figure is a replicate of Figure 6b and includes the name of each node (gene). The Spearman's Rho of two node generates the color and depth of each edge. The color (pink, yellow, and green) of node indicates each group (group α, β, and γ).
**Table S1.** Summary of training and testing datasets.
**Table S2.** The values of sensitivity, specificity, accuracy and balanced accuracy according each different number of selected features.
**Table S3.** Performance comparison based on the selected features from RF, XGBoost, AdaBoost and their ensemble.

**Data S1.** Gene information.

**Data S2.** Ranked Feature importance.
