# Supplementary material for: Artificial‐intelligence‐driven discovery of prognostic biomarker for sarcopenia
Source: J Cachexia Sarcopenia Muscle. 2021 Oct 26;12(6):2220–30. doi: 10.1002/jcsm.12840 (PMC8718042; doi:10.1002/jcsm.12840)
Supplement: Supplementary file 1 — Figure S1. Histogram for the number of available gene information. 39 subjects include approximately 16,100 gene information out of 17,339. 40 subjects include approximately 16,600 gene information. 39 subjects include approximately 16,700 gene information. Figure S2. Receiver operating characteristic curves (ROCs); four different models of RF, XGBoost, AdaBoost, and DSnet‐v1based on testing data. Figure S3. Scatter plots showing the relative expression of each gene in the healthy and sarcopenic elderly in the three different races. Figure S4. Gene network showing co‐expression of group α, β, and γ. This figure is a replicate of Figure 6b and includes the name of each node (gene). The Spearman's Rho of two node generates the color and depth of each edge. The color (pink, yellow, and green) of node indicates each group (group α, β, and γ). Table S1. Summary of training and testing datasets. Table S2. The values of sensitivity, specificity, accuracy and balanced accuracy according each different number of selected features. Table S3. Performance comparison based on the selected features from RF, XGBoost, AdaBoost and their ensemble. [file JCSM-12-2220-s003.docx]

Artificial-intelligence-driven discovery of prognostic biomarker for sarcopenia

# ^1^Heewon Chung^‡^, ^2,3^Yunju Jo^‡^, ^2,3^Dongryeol Ryu, ^3,4^ Changwon Jeong, ^3,5^Seong-Kyu Choe*, and ^1^Jinseok Lee*

^1^Department of Biomedical Engineering, College of Electronics and Information, Kyung Hee University, Yongin-si, Gyeonggi-do 17104, Republic of Korea

^2^Department of Molecular Cell Biology, Sungkyunkwan University School of Medicine, Suwon, 16419, Republic of Korea

^3^Sarcopenia Total Solution Center, Wonkwang University School of Medicine, Iksan, Jeonbuk 54538, Republic of Korea

^4^Medical Convergence Research Center, Wonkwang University, Iksan, 54538, Republic of Korea

^5^Department of Microbiology, and Institute of Wonkwang Medical Science, Wonkwang University School of Medicine, Iksan, Jeonbuk, 54538, Republic of Korea

^‡^ These authors contributed equally to this work

Corresponding author: S.-K. Choe (e-mail: seongkyu642@wku.ac.kr) and J. Lee (e-mail:  gonasago@khu.ac.kr).


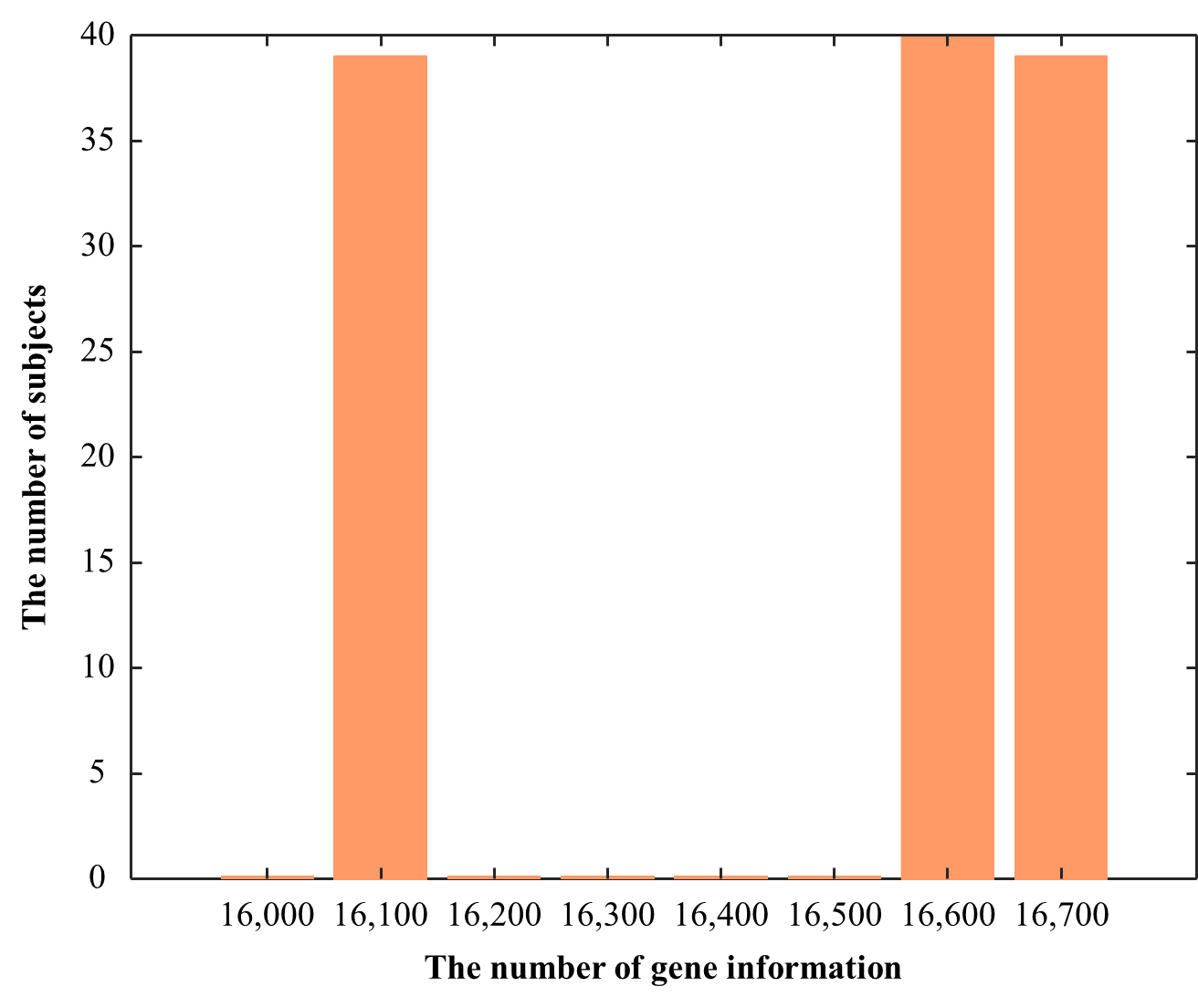


**Figure S1.** Histogram for the number of available gene information. 39 subjects include approximately 16,100 gene information out of 17,339. 40 subjects include approximately 16,600 gene information. 39 subjects include approximately 16,700 gene information.


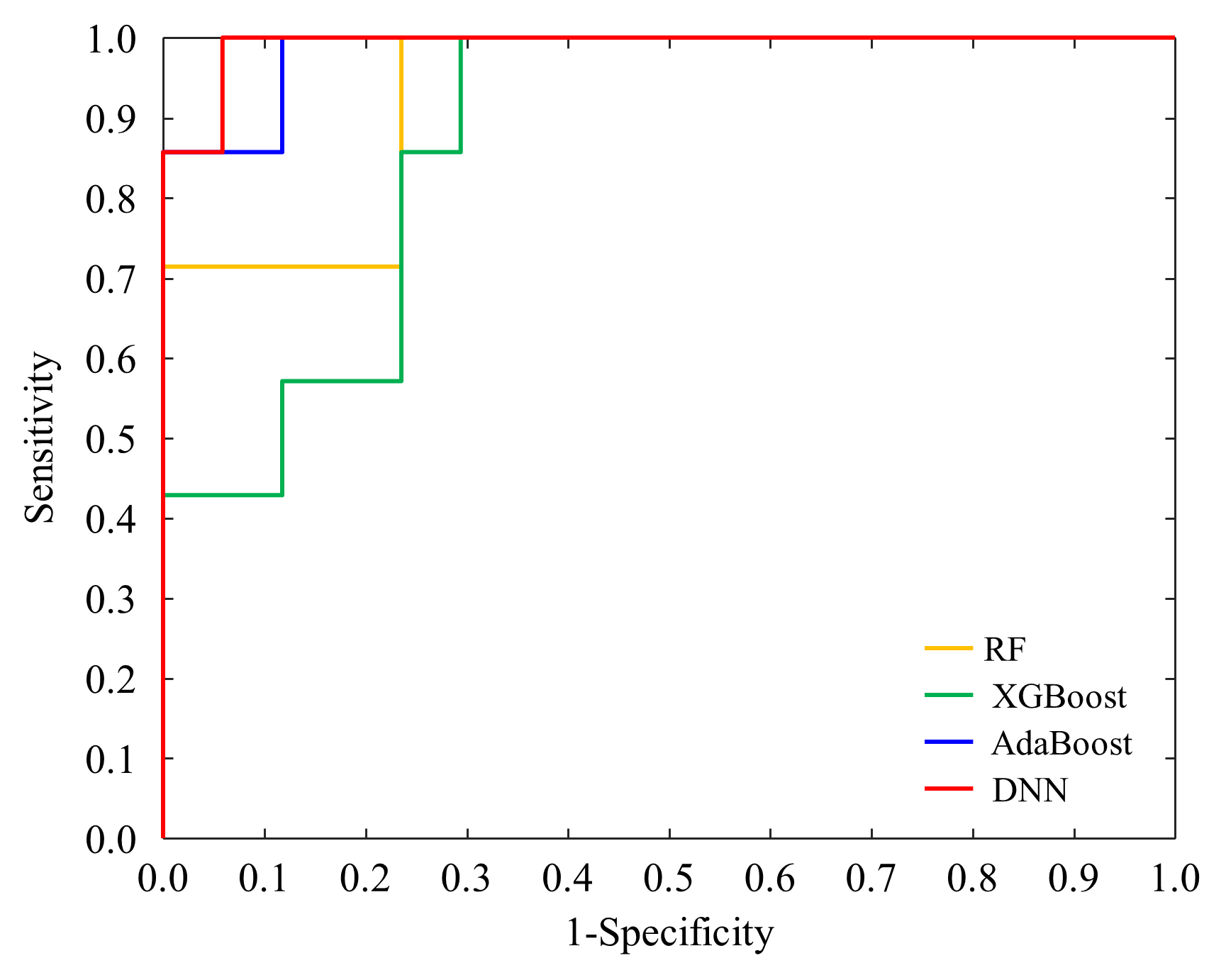


**Figure S2**. Receiver operating characteristic curves (ROCs); four different models of RF, XGBoost, AdaBoost, and DSnet-v1based on testing data.


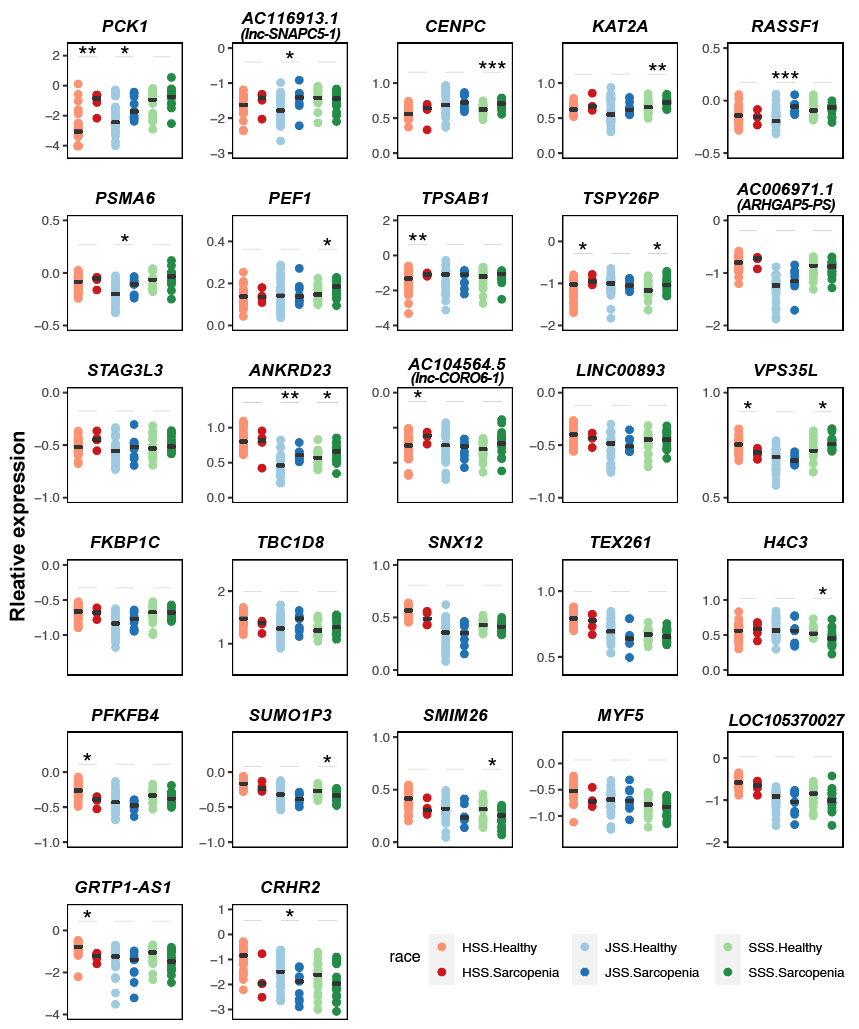


**Figure S3.** Scatter plots showing the relative expression of each gene in the healthy and sarcopenic elderly in the three different races. This figure is a modified version of Fig. 4a to display race-specific genes that are differentially expressed between healthy and sarcopenic elderly within a race. *P < 0.05, **P < 0.01, ***P < 0.001; P values calculated using two-way ANOVA followed by Tukey's multiple comparisons test.


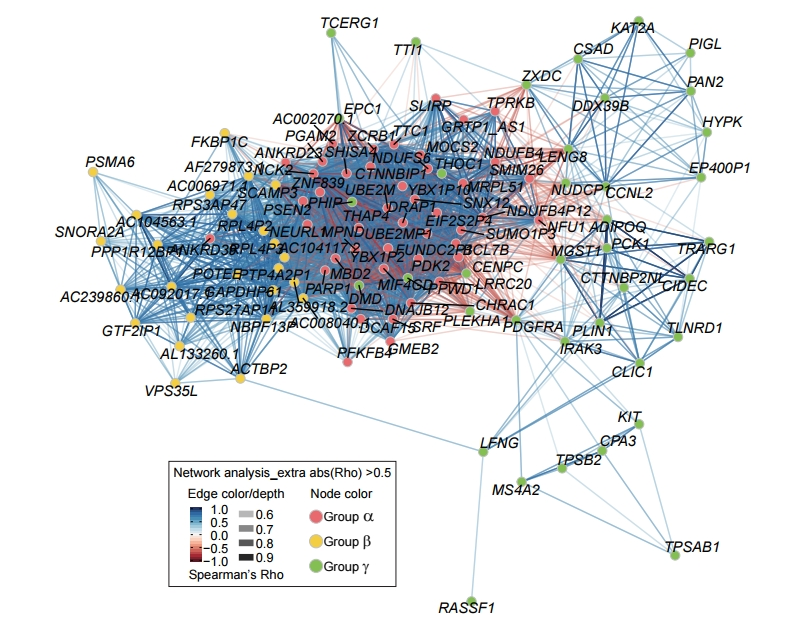


**Figure S4.** Gene network showing co-expression of group α, β, and γ. This figure is a replicate of Fig. 5b and includes the name of each node (gene). The Spearman's Rho of two node generates the color and depth of each edge. The color (pink, yellow, and green) of node indicates each group (group α, β, and γ).

**Table S1.** Summary of training and testing datasets.

| Study name | HSS | | JSS | | CSS | | Total | |
| --- | --- | --- | --- | --- | --- | --- | --- | --- |
|  | normal | sarcopenia | normal | sarcopenia | normal | sarcopenia | normal | sarcopenia |
| Train | 29 | 3 | 24 | 7 | 16 | 15 | 69 | 25 |
| Test | 7 | 1 | 6 | 2 | 4 | 4 | 17 | 7 |
| Total | 36 | 4 | 30 | 9 | 20 | 19 | 86 | 32 |

**Table S2.** The values of sensitivity, specificity, accuracy and balanced accuracy according each different number of selected features.

| The number of selected top features | TN | FP | FN | TP | Sensitivity | Specificity | Accuracy | Balanced Accuracy | AUROC |
| --- | --- | --- | --- | --- | --- | --- | --- | --- | --- |
| 1 | 11 | 58 | 15 | 10 | 0.4000 | 0.1594 | 0.2234 | 0.2797 | 0.5057 |
| 2 | 27 | 42 | 13 | 12 | 0.4800 | 0.3913 | 0.4149 | 0.4357 | 0.5210 |
| 3 | 39 | 30 | 11 | 14 | 0.5600 | 0.5652 | 0.5638 | 0.5626 | 0.5882 |
| 5 | 41 | 28 | 11 | 14 | 0.5600 | 0.5942 | 0.5851 | 0.5771 | 0.6639 |
| 6 | 47 | 22 | 10 | 15 | 0.6000 | 0.6812 | 0.6596 | 0.6406 | 0.6807 |
| 8 | 52 | 17 | 10 | 15 | 0.6000 | 0.7536 | 0.7128 | 0.6768 | 0.7983 |
| 9 | 55 | 14 | 11 | 14 | 0.5600 | 0.7971 | 0.7340 | 0.6786 | 0.7983 |
| 10 | 57 | 12 | 8 | 17 | 0.6800 | 0.8261 | 0.7872 | 0.7530 | 0.8067 |
| 12 | 59 | 10 | 7 | 18 | 0.7200 | 0.8551 | 0.8191 | 0.7875 | 0.8571 |
| 14 | 60 | 9 | 11 | 14 | 0.5600 | 0.8696 | 0.7872 | 0.7148 | 0.8067 |
| 15 | 63 | 6 | 8 | 17 | 0.6800 | 0.9130 | 0.8511 | 0.7965 | 0.8655 |
| 18 | 64 | 5 | 7 | 18 | 0.7200 | 0.9275 | 0.8723 | 0.8238 | 0.8824 |
| 20 | 64 | 5 | 5 | 20 | 0.8000 | 0.9275 | 0.8936 | 0.8638 | 0.9244 |
| **27** | **67** | **2** | **3** | **22** | **0.8800** | **0.9710** | **0.9468** | **0.9255** | **0.9748** |
| 34 | 66 | 3 | 3 | 22 | 0.8800 | 0.9565 | 0.9362 | 0.9183 | 0.9496 |
| 39 | 66 | 3 | 4 | 21 | 0.8400 | 0.9565 | 0.9255 | 0.8983 | 0.9328 |
| 42 | 67 | 2 | 4 | 21 | 0.8400 | 0.9710 | 0.9362 | 0.9055 | 0.9412 |
| 48 | 65 | 4 | 6 | 19 | 0.7600 | 0.9420 | 0.8936 | 0.8510 | 0.9244 |
| 57 | 63 | 6 | 10 | 15 | 0.6000 | 0.9130 | 0.8298 | 0.7565 | 0.8235 |
| 794 | 63 | 6 | 11 | 14 | 0.5600 | 0.9130 | 0.8191 | 0.7365 | 0.8067 |
| 805 | 65 | 4 | 9 | 16 | 0.6400 | 0.9420 | 0.8617 | 0.7910 | 0.8571 |
| 857 | 63 | 6 | 9 | 16 | 0.6400 | 0.9130 | 0.8404 | 0.7765 | 0.8319 |
| 882 | 48 | 21 | 10 | 15 | 0.6000 | 0.6957 | 0.6702 | 0.6478 | 0.7647 |
| 889 | 44 | 25 | 8 | 17 | 0.6800 | 0.6377 | 0.6489 | 0.6588 | 0.7731 |
| 15,538 | 12 | 57 | 15 | 10 | 0.4000 | 0.1739 | 0.2340 | 0.2870 | 0.5126 |

**Table S3.** Performance comparison based on the selected features from RF, XGBoost, AdaBoost and their ensemble

| Feature importance | TN | FP | FN | TP | Sensitivity | Specificity | Accuracy | Balanced Accuracy |
| --- | --- | --- | --- | --- | --- | --- | --- | --- |
| RF | 13 | 4 | 1 | 6 | 0.8571 | 0.7647 | 0.7917 | 0.8109 |
| XGBoost | 15 | 2 | 0 | 7 | 1.0000 | 0.8824 | 0.9167 | 0.9412 |
| AdaBoost | 17 | 0 | 1 | 6 | 0.8571 | 1.0000 | 0.9583 | 0.9286 |
| Ensemble | 16 | 1 | 0 | 7 | 1.0000 | 0.9412 | 0.9583 | 0.9916 |
